# Management of Hypertension in the Obese Pregnant Patient

**DOI:** 10.1007/s11906-019-0927-x

**Published:** 2019-03-26

**Authors:** Christina Coroyannakis, Asma Khalil

**Affiliations:** 1 Fetal Medicine Unit, St George’s Hospital, St George’s University of London, Cranmer Terrace, London, SW17 0RE UK; 2 Vascular Biology Research Centre, Molecular and Clinical Sciences Research Institute, St George’s University of London, London, UK; 3 Fetal Medicine Unit, Department of Obstetrics & Gynaecology, St George’s University Hospitals NHS Foundation Trust, Blackshaw Road, London, SW17 0QT UK

**Keywords:** Hypertension management, Obese, Pregnant

## Abstract

**Purpose of Review:**

To evaluate recent developments in the management of hypertension in obese pregnant women.

**Recent Findings:**

The mainstay of management targets prevention of hypertension with preconception counselling, entering pregnancy with a lower BMI, limiting weight gain, and taking low-dose aspirin to prevent pre-eclampsia from before 16 weeks’ gestation. There are conflicting results regarding the use of metformin in reducing hypertensive disease, but there is a high probability that it has a role to play. Clinical trials are in progress examining the use of statins in preventing pre-eclampsia, with promising results from pre-clinical trials. Home blood pressure monitoring may be helpful in diagnosing and monitoring the control of hypertension.

**Summary:**

The most protective interventions against hypertensive disease in obese pregnant women are entering pregnancy at a lower BMI, avoiding inter-pregnancy weight gain, and taking low-dose aspirin during pregnancy. Further research is needed around the use of metformin, statins, and home blood pressure monitoring.

## Introduction

Obesity is associated with an increasingly huge impact on healthcare globally. Its effect in pregnancy has both immediate and long-term implications on the maternal and child health. The prevalence of obesity in the UK population is increasing, and it is estimated that approximately 1 in 20 pregnant women have a body mass index (BMI) of over 35 kg/m^2^ [1]. This not only represents a huge challenge to maternity services, but also represents a sizeable financial burden at a time when healthcare funding is continually being cut [2]. There has been a concerted effort to try to reduce obesity levels in the general population given the mounting evidence that it is associated with multiple co-morbidities, including diabetes, cardiovascular disease, hypertension, gastro-intestinal disease, and psychiatric morbidities [3]. There is now also a growing body of evidence that, during pregnancy, there is a significant effect on the offspring’s health, increasing their likelihood of stillbirth, requiring admission to the neonatal unit, being large for gestational age, and being obese themselves as children [1, 2]. Efforts have focused on preconception counselling, limiting weight gain during pregnancy, exploring pharmacological options, and improving blood pressure monitoring in order to prevent and better manage hypertensive disorders associated with obese pregnant women.

## Obesity in Pregnancy

**Fig. 1:**
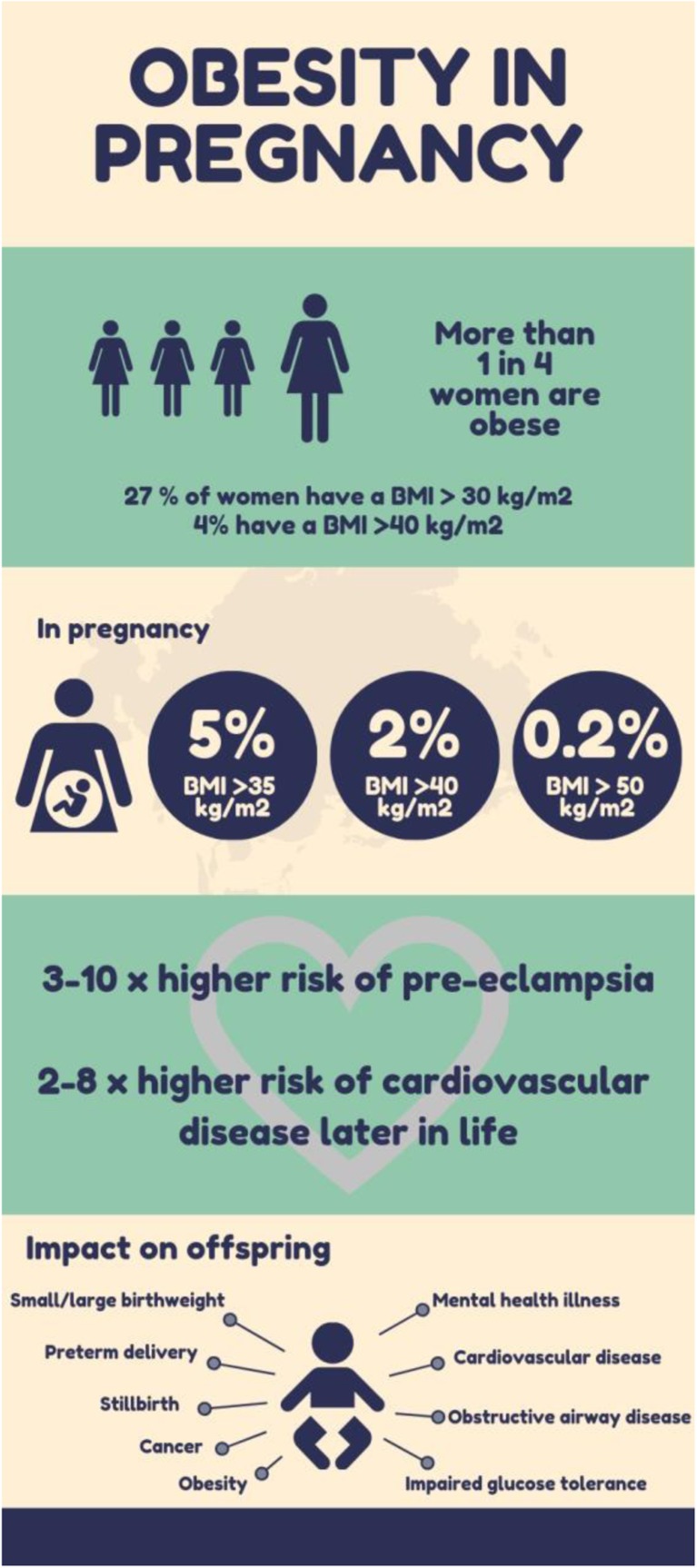
The prevalence and impact of obesity in pregnancy

In the most recent Health Survey for England, the prevalence of adult obesity was 27% in women, with 4% of women being morbidly obese. This means that their body mass index, calculated by taking into account their height and their weight, is above 30 kg/m^2^ or above 40 kg/m^2^, respectively [4]. This translates into an increasing number of obese women falling pregnant and is associated with a higher prevalence of co-morbid conditions and both maternal and fetal complications (Fig. 1) [1].

Morbidity and mortality are incrementally higher with each increase in BMI category, subdivided into Class I (> 30 kg/m^2^), Class II (> 35 kg/m^2^), Class III or morbid obesity (> 40 kg/m^2^), and super-morbid obesity (> 50 kg/m^2^). The prevalence of women with a BMI above 35 kg/m^2^ who gave birth beyond 24 weeks gestation is 5%, with 2% being in the Class III category and 0.2% being the super-morbid obesity category [1, 3, 4]. Women in the higher BMI categories had a significant increase in the incidence of pregnancy-induced hypertension, pre-eclampsia, and severe pre-eclampsia [1]. Systematic reviews have concluded that there is a direct relationship between obesity and pre-eclampsia with a 3 to 10 times increase in the risk in obese women [5]. This, in turn, impacts negatively on the likelihood of spontaneous vaginal delivery without instrumentation and increases the rate of caesarean section, postpartum haemorrhage, longer hospital stays, neonatal admissions, stillbirths, and infant death [1]. Longer term, there is a life-long risk of cardiovascular disease in both the mother and the offspring. This includes hypertension, ischaemic heart disease, and stroke with some studies finding a two to eightfold increase in the risk of developing cardiovascular disease following a pregnancy complicated by pre-eclampsia [5, 6]. It is clear that a holistic and multi-faceted approach is key in ensuring the optimal management of the obese hypertensive pregnant woman, in order to reduce the immediate and long-term health implications for both the mother and offspring.

## Preconception Counselling

Cardiovascular disease is associated with a number of modifiable risk factors including smoking, hypertension, hyperlipidaemia, sedentary lifestyle, and insulin resistance [6, 7]. Women of childbearing age contemplating pregnancy represent a window of opportunity to target these risk factors. By optimising their health prior to pregnancy, they benefit from improved fertility, more uncomplicated pregnancies and deliveries, healthier offspring, and better long-term health, both physical and mental [5]. Many women are unaware of the implications obesity has on their health and their fertility. Educating them about the lifestyle changes that can optimise their chances of a successful pregnancy can aid them in making informed decisions about timing their conception. The aim of improving their fertility can act as a positive driving force to introduce and maintain those lifestyle changes [5, 8, 9].

The main objective is to encourage women to achieve a BMI within the normal range of 18.5–24.9 kg/m^2^ [3, 10]. However, a reduction in pre-pregnancy BMI of even 10% has been shown to reduce the risk of pre-eclampsia by 10% [11]. The timing of this counselling needs to be explored as this is information that potentially needs to be disseminated from a young age in order to tackle rising obesity rates well before the point of falling pregnant [10]. There is an association between weight reduction around the time of conception and adverse perinatal outcomes such as low birth weight, preterm delivery, and longer-term health implications in offspring like cardiovascular disease, impaired glucose tolerance, obstructive airways disease, schizophrenia, and breast cancer as adults [10, 12]. Therefore, women undergoing bariatric surgery are encouraged to wait 1–2 years before falling pregnant in order to avoid the period of most rapid weight loss and malabsorption [10].

Unfortunately, the availability of preconception services and women’s engagement with these services is lacking. According to the organisational survey of UK maternity units, only 6% of obstetric units offer preconception services to women with a BMI over 30 kg/m^2^ [1]. Even where the services exist, less than half of the women, it is designed to reach, engage with them [9]. This does not take into account the advice given in the primary care setting, but this does highlight the need for more opportunities to provide individualised preconception counselling to obese women planning to get pregnant.

## Weight Gain

Many studies have focused on the effects of weight gain during pregnancy and during the inter-pregnancy interval. Weight gain during pregnancy has been associated with an increased risk of developing gestational diabetes, large for gestational-age babies, insulin resistance in the offspring, and stillbirths, but also can increase the BMI in the postpartum period leading to increased BMI in subsequent pregnancies [10, 13, 14].

When looking at hypertensive disorders, excessive weight gain during pregnancy has been associated with increased risks of developing gestational hypertension and pre-eclampsia [15, 16]. The difficulty arises that although women are advised to avoid excessive gestational weight gain, no intervention has currently been found that significantly reduces maternal and fetal adverse outcomes. The UPBEAT study looked at the clinical outcomes of obese pregnant women after supporting them with health trainer–led sessions to improve diet and physical activity. Unfortunately, these behavioural interventions did not significantly reduce the risk of pre-eclampsia or any other adverse outcomes [17]. A similar study in Australia found that support through a dietician and setting goals for dietary change and exercise did not significantly impact the incidence of hypertensive disease in pregnancy either [18].

There is now more focus on trying to avoid weight gain in between pregnancies. It seems that entering pregnancy with a healthier BMI is associated with better immediate and long-term outcomes [5, 10, 14, 16]. The inter-pregnancy weight gain is directly linked to an increased risk of developing hypertensive disease in subsequent pregnancies. For gestational hypertension and for pre-eclampsia, the risk increases with increasing BMI with a relative risk of 2.24 and 1.64 for a BMI increase of 3 kg/m^2^ respectively (Table 1) [14]. It is also related to an increased risk of hypertensive disease later in life [6, 10]. It is therefore essential to target women in the postnatal period in order to educate them and support them to achieve their weight loss goals and not carry the weight through to the next pregnancy or later in life [16].

**Table 1:** Relative risks (95% confidence intervals) of maternal hypertensive disease in the second pregnancy based on increase in pre-pregnancy body mass index (BMI) in women who are nulliparous at baseline without hypertensive disease in the first pregnancy (*n* = 42,399) [14]

Outcome in the second pregnancy	BMI change (in kg/m^2^)			
	< 1	1 to < 2	2 to < 3	> 3
Pre-eclampsia (*n* = 464)				
Unadjusted	1.13	1.11	1.04	2.24
	(0.84–1.50)	(0.84–1.48)	(0.72–1.49)	(1.78–2.82)
Adjusted	0.84	0.98	0.87	1.64
	(0.63–1.13)	(0.74–1.31)	(0.60–1.26)	(1.27–2.11)
Gestational hypertension (*n* = 587)				
Unadjusted	1.07	1.35	1.20	2.90
	(0.81–1.40)	(1.05–1.72)	(0.87–1.65)	(2.38–3.53)
Adjusted	0.78	1.24	1.03	2.24
	(0.59–1.03)	(0.97–1.59)	(0.75–1.42)	(1.82–2.76)

Adjusted for maternal race, inter-pregnancy interval, maternal age, marital status, smoking, and alcohol use during the second pregnancy, pre-pregnancy BMI, gestational diabetes mellitus, pre-eclampsia, and gestational hypertension in the first pregnancy

## Aspirin

The use of aspirin in pregnancy has been put forward in order to try to prevent a number of adverse pregnancy outcomes, including preterm deliveries, intrauterine fetal growth restriction, miscarriages, and stillbirths. The most common use of aspirin in pregnancy is to reduce the risk of pre-eclampsia or to delay its onset [16, 19]. Given the association of obesity with all of the aforementioned adverse outcomes, aspirin has been recommended for use in women with a BMI over 35 kg/m^2^ who have at least one other moderate risk factor for pre-eclampsia [20].

The optimal dose of aspirin is yet to be ascertained, with an initial recommendation of having 75 mg once a day [21]. It was found that there was a 10% reduction in the risk of pre-eclampsia when taking a dose of at least 75 mg once a day and that this benefit was lost when aspirin was started after 20 weeks gestation [22, 23]. Further to this, it was noted that the maximal beneficial effect was obtained if aspirin was started before 16 weeks’ gestation [24]. A dose-dependent benefit was also observed and, as a result, the ASPRE study was set up across 13 countries in order to determine the effect of 150 mg of aspirin daily started at between 11 and 14 weeks’ gestation and continued until 36 weeks’ gestation. There was a significant reduction in the occurrence of preterm pre-eclampsia in women with singleton pregnancies who were found to be at high risk of pre-eclampsia with an odds ratio of 0.38 (see Table 2). Specifically, there was a reduction of new-onset hypertension with proteinuria. There were, however, no significant differences in the development of preterm or term gestational hypertension without proteinuria [25].

**Table 2:** Outcomes for hypertensive disease in trial group [25]

Outcome	Aspirin group (*n* = 798)	Placebo group (*n* = 822)	Odds ratio (95% or 99% CI)*
Primary outcome: pre-eclampsia at < 37 weeks gestation	13 (1.6)	35 (4.3)	0.38 (0.20–0.74)
Secondary outcomes: pre-eclampsia at < 34 weeks gestation	3 (0.4)	15 (1.8)	0.18 (0.03–1.03)
Gestational hypertension at < 37 weeks gestation	8 (1.0)	7 (0.9)	1.19 (0.31–4.56)
Gestational hypertension at < 34 weeks gestation	2 (0.3)	2 (0.2)	1.02 (0.08–13.49)

*Confidence interval for primary outcomes 95% and secondary outcomes 99%

The values represent the actual numbers and those in the brackets represent percentage (%)

The safety of the use of aspirin has been well documented with no negative maternal or perinatal outcomes and only 10% of women experiencing gastro-intestinal discomfort [22]. It is therefore now a widely used and widely recommended treatment for the prevention of pre-eclampsia.

## Metformin

There is conflicting data supporting the use of metformin as a treatment for hypertensive disorders in pregnancy. It is widely used to treat gestational diabetes and is considered safe for use in pregnancy [26]. Metformin is a drug that increases tissue sensitivity to insulin, thus lowering glucose levels in the blood. It inhibits gluconeogenesis in the liver and improves uptake of glucose into the skeletal muscles and fat cells, but has also been linked with improved cardiovascular function and is nephro-protective [27].

The EMPOWaR study randomised non-diabetic women with a BMI over 30 kg/m^2^ to receive metformin initiated at 12–16 weeks’ gestation in order to determine its effect on fetal birth weight. As a secondary outcome, it looked at the incidence of adverse maternal outcomes, including pregnancy-induced hypertension and pre-eclampsia. There was no statistically significant reduction in the women receiving metformin [28]. Conversely, a similar study randomising women with a BMI over 35 kg/m^2^ to receive 3 g of metformin a day from 12 to 18 weeks’ gestation until delivery, not only found a statistically significant lower incidence of pre-eclampsia but also reduced weight gain during pregnancy [26].

A Cochrane review on the use of metformin in obese pregnant women conducted in 2017 included these two studies and only one other study from Egypt, emphasising the paucity of studies addressing this topic. They were able to include data on 1099 participants and stated that metformin may make little or no difference in the context of prevention of gestational hypertension or pre-eclampsia in obese pregnant women [29]. Since then, a systematic review and meta-analysis has been published including 15 studies. Again, with regard to obese pregnant women, no statistically significant difference was demonstrated in treating hypertensive diseases in pregnancy with the use of metformin. The meta-analysis was able to extrapolate that there was a high probability of over 90% that metformin has a beneficial effect in preventing pre-eclampsia, gestational hypertension, and any hypertensive disease in pregnancy, when compared to placebo or other treatments. However, the low quality of evidence and the heterogeneity of the studied outcomes and results made it difficult to make recommendations for wider use [30]. More research is required in order to fully understand the impact that metformin can have on its own or in combination with other treatments.

## Statins

Statins have long been used in the primary and secondary prevention of atherosclerotic cardiovascular disease [31]. Their use in women of childbearing age is increasing with the rise in obesity and accompanying rise in cholesterol levels. Although limited data is available with regard to their use and safety in pregnancy, inadvertent exposure is occurring when unplanned pregnancies occur [32].

Earlier studies found that there was an increased risk of teratogenic effects following exposure to statins. This has not been supported by more recent studies, and in fact, statins have been shown to be safe and have therapeutic effects in the context of pre-eclampsia in pre-clinical trials [32, 33]. Pravastatin in particular has been shown to have anti-inflammatory, anti-thrombogenic, and antioxidant effects resulting in lower pre-eclampsia-related markers and improved endothelial function [33]. The StAmP study is a multicentre UK trial that recruited 62 women with singleton pregnancies diagnosed with pre-eclampsia between 24 and 32 weeks gestation in order to determine the clinical benefits of pravastatin in pre-eclampsia. A publication with their outcome data is still awaited [34]. A similar study in the USA, titled “Pravastatin for Prevention of Preeclamsia” is in its final stages [35]. The outcomes from both of those studies will hopefully help to guide the future use of statins in pregnancy.

## Home Blood Pressure Monitoring

Historically, monitoring for hypertensive disease in pregnancy has relied upon women’s regular attendance at antenatal visits, with blood pressure checks at each visit. In women identified as being at high risk of developing pre-eclampsia, more frequent blood pressure measurements are recommended, but no timings or intervals are specified [36]. In primary care settings, the use of home blood pressure monitoring devices in the management of hypertension has been incorporated as part of a strategy to involve the patient in the management of their own care. It has been used to good effect resulting in significantly lower blood pressure readings compared to titration of antihypertensive medication based on clinic readings alone [37].

Extended to the pregnant population at risk of or having already developed hypertensive disease, a home blood pressure monitoring strategy can help to manage women more accurately without increasing their risk of adverse outcomes. As a secondary effect, it may also reduce the number of visits to hospital in the context of outpatient monitoring and provide a more acceptable way for women to monitor their blood pressure [38]. There is evidence that home blood pressure monitoring is safe provided the reference ranges for target blood pressures are lower than in the clinic setting [37, 38]. In obese pregnant women, the use of an appropriate sized cuff is important in ensuring accurate blood pressure readings [20].

Home blood pressure monitoring can not only enable closer and more accurate monitoring, but can also help to enable appropriate diagnosis and initiation of antihypertensive medication. According to the NICE guidance, the first line medication in the treatment of hypertension in pregnancy is labetalol, followed by nifedipine and methyldopa [39]. There is no particular mention regarding any difference in the choice of antihypertensive therapy in the obese pregnant woman. With the adjunct of a home blood pressure monitor that has been validated for use in pregnancy and a specially designed smartphone app, women’s blood pressure can be monitored remotely and antihypertensive treatment can be titrated appropriately without compromising safety [38]. This innovative pathway has also been shown to be cost saving in UK NHS healthcare setting [40].

## Discussion

A range of different methods to tackle hypertension in obese pregnant women have been put forward, some are more effective than others. Insufficient resources have targeted the provision of a holistic preconception counselling service. In the few places where this service is provided, it is not being accessed enough and women are not attending, as it is associated with the stigma of over-medicalisation and the fear of pregnancy complications [9]. Pre-pregnancy counselling represents a unique window of opportunity to address weight issues in order to enter a pregnancy in better health with the prospect of better outcomes for both mother and baby [13, 16]. It appears that limiting weight gain during the pregnancy itself is too little of an intervention too late in the process [17, 18].

A number of pharmacological approaches are being introduced. Aspirin at a dose of 150 mg once a day initiated before 16 weeks gestation is by far the drug with the most evidence of being effective at reducing preterm pre-eclampsia [25]. Metformin has some promise but larger studies are needed in the context of obesity in order to elucidate whether it truly is beneficial in reducing hypertensive disease in pregnancy [30]. The evidence from clinical trials for the use of statins is awaited and also holds some promise based on pre-clinical trials [33].

With the advent of smartphones and evolving technologies, home blood pressure monitoring may become the mainstay of the management of hypertensive diseases both during the pregnancy and postnatally in the community [37, 38]. This will hopefully achieve better blood pressure control and reduce the need for as many hospital visits without jeopardising maternal and fetal outcomes [38].

It is clear that with a growing obese population, determining the most effective treatment options and lifestyle recommendations will have increasing importance and is likely to influence care pathways and service provision.

## Conclusions

Obesity is a growing health concern that is affecting an increasing number of pregnancies and presents a multi-faceted challenge to providers of maternity care. It is associated with multiple co-morbidities and has a direct link with hypertensive disease of pregnancy. Individualised, holistic, preconception care is lacking and needs to be more readily available. Pregnancies identified as high risk for developing hypertensive disease should be more closely monitored, possibly with the help of home blood pressure monitoring, and low-dose aspirin should be started before 16 weeks’ gestation. Further research needs to be undertaken to determine the role that metformin and statins have on reducing the risk of hypertensive disease in pregnancy. Close ties between hospital and community care, in the preconception, antenatal, and postnatal periods, can support women to limit weight gain, make informed choices and time first and subsequent pregnancies so that they have optimised their health and reduced their BMI.
